# Phase Equilibria, Solidified Microstructure, and Hydrogen Transport Behaviour in the V-Ti-Co System

**DOI:** 10.3390/membranes13090790

**Published:** 2023-09-12

**Authors:** Erhu Yan, Zhijie Guo, Limin Jia, Yihao Wang, Shuo Zhang, Tangwei Li, Yongjin Zou, Hailiang Chu, Huanzhi Zhang, Fen Xu, Lixian Sun

**Affiliations:** 1 Guangxi Key Laboratory of Information Materials, Guilin University of Electronic Technology, Guilin 541004, China; guozhijie000002@163.com (Z.G.); wabgyihao0034@163.com (Y.W.); zhangshuo_0902@163.com (S.Z.); litangwei_1222@163.com (T.L.); zouy@guet.edu.cn (Y.Z.); chuhailiang@guet.edu.cn (H.C.); zhanghuanzhi@guet.edu.cn (H.Z.); xufen@guet.edu.cn (F.X.); sunlx@guet.edu.cn (L.S.); 2 Hebei Key Laboratory of Material Near-Net Forming Technology, School of Materials Science and Engineering, Hebei University of Science and Technology, Shijiazhuang 050018, China

**Keywords:** V-Ti-Co alloy, phase diagram, membranes, hydrogen permeation

## Abstract

At present, the V-Ti-Co phase diagram is not established, which seriously hinders the subsequent development of this potential hydrogen permeation alloy system. To this end, this article constructed the first phase diagram of the V-Ti-Co system by using the CALculation of PHAse Diagrams (CALPHAD) approach as well as relevant validation experiments. On this basis, hydrogen-permeable V_x_Ti_50_Co_50−x_ (x = 17.5, 20.5, …, 32.5) alloys were designed, and their microstructure characteristics and hydrogen transport behaviour were further studied by XRD, SEM, EDS, and so on. It was found that six ternary invariant reactions are located in the liquidus projection, and the phase diagram is divided into eight phase regions by their connecting lines. Among them, some alloys in the TiCo phase region were proven to be promising candidate materials for hydrogen permeation. Typically, V_x_Ti_50_Co_50−x_ (x = 17.5–23.5) alloys, which consist of the primary TiCo and the eutectic {bcc-(V, Ti) and TiCo} structure, show a high hydrogen permeability without hydrogen embrittlement. In particular, V_23.5_Ti_50_Co_26.5_ exhibit the highest permeability of 4.05 × 10^−8^ mol H_2_ m^−1^s^−1^Pa^−0.5^, which is the highest value known heretofore in the V-Ti-Co system. The high permeability of these alloys is due in large part to the simultaneous increment of hydrogen solubility and diffusivity, and is closely related to the composition of hydrogen permeable alloys, especially the Ti content in the (V, Ti) phase. The permeability of this alloy system is much higher than those of Nb-TiCo and/or Nb-TiNi alloys.

## 1. Introduction

In recent years, extensive research was conducted on non-Pd hydrogen permeable alloys due to the high price (~USD 90 per gram) and scarce resources of Pd-based alloys (Pd-Ag [1], Pd-Au [2], etc.), one major aspect of this is group 5B metals, such as V, Nb, and Ta [3]. To overcome the hydrogen embrittlement issue of these pure metals, it is usually necessary to dope them with other late transition metals (Ni, Co, Mo, etc.) to form binary or ternary alloys [4,5]. In the past decade, significant efforts were made in the experimental study of multi-phase Nb- or V-based hydrogen permeable alloys by Dolan [4,6], Fleury [6], Yukawa [7], Aoki [8], and Nishimura [9,10]. Of these alloys, Nb–TiCo duplex alloys, e.g., Nb_30_Ti_35_Co_35_, were demonstrated to be promising candidates for new hydrogen permeation membranes by Ishikawa et al. [11]. Additionally, they also found that these alloys, which contain the B2–TiCo compound and the bcc-(Nb, Ti) solid solution, not only have high permeability (using Pd as a reference) but also excellent resistance to hydrogen embrittlement. Within the range of hydrogen permeation pressure of 0.1 to 0.5 MPa, the hydrogen dissolution reaction into these Nb- or V-based ternary alloys [12] follows the pseudo-Sieverts’ law, *C* = *K*⋅*P*^1/2^ + α. This is different from Pd-based alloy, which satisfies the Sieverts’ law (*C = K⋅P*^1/2^). Generally, hydrogen dissolution or absorption is a dynamic process that changes over time, and this complete process is difficult to dynamically track. Therefore, using molecular dynamics simulations (MDS) [13] to carry out related research is also one of the current research hotspots.

Recently, this system was further investigated by our research group, and the alloy composition range suitable for hydrogen permeation was determined for the first time [14]. Afterwards, combined with a melt spinning technique, more promising Nb-Ti-Co amorphous membranes were successfully prepared. These membranes show a high H_2_ flux of 15.55 cc H_2_ cm^−2^ min^−1^ and can work continuously for up to 112 h (see Ref. [14]). According to the periodic table, both V and Nb metals belong to the 5B group and have similar crystal structures and physical/chemical properties. Similar to the Nb-TiCo alloy system studied earlier [11,14], it is strongly expected that there are also suitable alloy compositions in the V-Ti-Co system, which has good performance with respect to hydrogen permeation. However, due to the complex interactions of multi-components, little research was conducted on the V-Ti-Co phase diagram, and currently, there are only reports on isothermal sections at 800 °C, 1000 °C, 1100 °C, and 1200 °C in the literature [15]. Furthermore, it is currently unclear whether there are dual-phase (bcc-V + TiCo) alloys used for hydrogen permeation in this system. 

Considering these aspects, the purpose and tasks of the present work are as follows: (1) to establish the equilibrium phase diagram of the V-Ti-Co system using the CALPHAD approach and obtain important solidification parameters, such as the types and quantities of the invariant reaction, the number and boundaries of phase zones, phase equilibrium reaction temperature, etc., (2) to evaluate the accuracy of the calculated V-Ti-Co phase diagram by means of Thermo-calc software based on the solidification experimental data of various alloys, and (3) to develop and explore alloys with excellent hydrogen transport performance, especially high permeability, in the newly established V-Ti-Co system. 

## 2. Experimental and Numerical Procedures

### 2.1. Materials and Methods

In this work, V-Ti-Co alloys for experimental research were fabricated through a vacuum non-consumable arc melting method, and the purity of raw materials (Beijing Dream Material Technology Co., Ltd., Beijing, China) was greater than 99.95%. The selection of alloy composition is mainly based on two considerations. One is to verify the accuracy of the calculated phase diagram, such as V_35_Ti_32.5_Co_32.5_, V_50_Ti_25_Co_25_, and V_30_Ti_27_Co_43_ alloys, while the other is used to explore new hydrogen permeation alloys. After weighing, cleaning, and vacuum drying, button shape ingots were prepared, and they were remelted five times to improve uniformity. Then, disc-shaped samples (*Φ* 16 × 0.7 mm) were cut from the centre of the ingots by using electric spark wire cutting technology. After polishing, the sample was kept in a vacuum state and a thin layer of pure Pd (purity = 99.99%) with a thickness of approximately 200 nm was deposited on its surface using a DC magnetron sputtering system at 573 K. Next, the sample is activated in an argon atmosphere for about 30 min. The chamber pressure was 5 × 10^−1^ Pa. The purpose of the Pd plating film is to enhance the catalytic activity of the composite membrane. Thus, the Pd film was involved in the hydrogen permeation experiment. The microstructure was characterized by a scanning electron microscope (SEM, MAIA3) operated at 200 kV. Correspondingly, the phase component was determined by energy-dispersive X-ray spectroscopy. The crystal structure before and after hydrogenation was analysed by X-ray diffraction (XRD, Rigaku D/MAX2550) with Cu-K*α* radiation (*λ* = 1.5418 Å). 

The hydrogen permeability of all the samples was measured using conventional gas permeation techniques. The test parameters were as follows: the upstream pressure (*P*_u_) of the membrane was 0.1–0.5 MPa, while the downstream pressure (*P*_d_) was maintained at 0.1 MPa. In other words, the value of *P*u was increased to 0.5 MPa by 0.05 MPa intervals. The initial testing temperature was 523 K. Then, the measurements were repeated at 573 K, 623 K, and 673 K, respectively. The membrane thickness (*L*) and effective hydrogen permeation area(*s*) were 0.68 mm and ~113 mm^2^, respectively. Finally, after measuring the H_2_ flux (*J*), linear fitting was performed on (*J* × *L*) and ($P_{u}^{0.5}$ and $P_{d}^{0.5}$), and the slope is the hydrogen permeability. The reason of multiplying flux (*J*) and thickness (*L*) is to eliminate the influence of membrane thickness when calculating the hydrogen permeability.

(1) $$J=\frac{\Phi \Delta P^{0.5}}{L}=\frac{\Phi (P_{u}{}^{0.5}-P_{d}{}^{0.5})}{L}$$

The meanings represented by unconventional symbols or abbreviations in all equations in this work can be seen in the abbreviation list. For more detailed information, please refer to our recent work in references [16,17,18]. 

### 2.2. Phase Diagram Calculation Model and Algorithm

#### 2.2.1. Thermodynamic Model

Table 1 summarises the available parameters and relevant thermodynamic data of the V-Ti-Co system. Overall, this alloy system contains three different types of phases, such as unary phases, solution phases, and intermetallic compounds. Each type is different and requires separate calculations. For pure elements in the V-Ti-Co system, their Gibbs energy G*_i_*(*T*) at 298.15 K and 100 kPa can be obtained by the following equation [19]:

(2) $$G_{i}(T)-H_{i}^{SER}=A+BT+CT\ln T+DT^{2}+ET^{-1}+IT^{7}+JT^{-9}$$

As for the solution phases (e.g., liquid, bcc-V, etc.), subregular solution models are referenced, and thus their Gibbs free energies (*G^Φ^_m_*) can be expressed by the following mathematical equation [21]:

(3) Gmφ−Gexφ=∑i=V,Ti,Coxi0⋅Giφ+R⋅T∑i=V,Ti,Coxi⋅lnxiφ+1T∑ixiTiφ+xixj∑m=onTmijφ(xi−xj)m

In Equation (3), the second term (*^ex^G*) at the left end is usually obtained through Redlich Kister polynomials [22,23]. $\sum_{i=V,Ti,Co}x_{i}^{0}\cdot G_{i}^{\varphi }$ is the mechanical mixing of V, Ti, or Co. Additionally, the intermetallic compounds, such as TiCo and TiCo_2_, etc., used the model reported by Wu et al. [24]. Due to the small composition range in the Ti-Co binary phase diagram, Ti_2_Co is approximately represented as a stoichiometric compound.

#### 2.2.2. Calculation Algorithm

**Figure 1:**
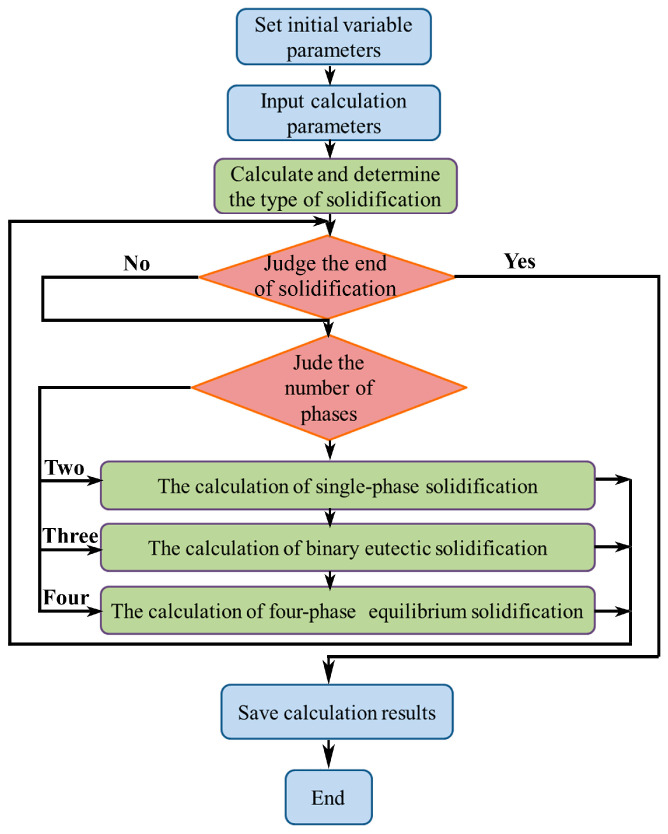
The algorithm flow chart of the solidification path for V-Ti-Co alloys.

In the simulation calculation process of the solidification path of the alloys, it is necessary to couple with our previously proposed unified micro-segregation model [25]. After inputting the initial parameters, each iteration calculation requires accessing the PKP database in Thermo-Calc software (v2019a) to obtain the corresponding thermodynamic data, which is then brought into the model for subsequent calculations (see Figure 1). The initialization parameters, such as solidification/cooling rates, step length, and so on, are shown in Table 1. Afterwards, the solidification type of the alloy is obtained by comparing the number of phases. In single-phase or binary eutectic solidification, the phase volume fraction (*f*_s_) is the control variable during the calculation process. In comparison, the temperature, *T*, is selected as the control variable during the four-phase equilibrium solidification, such as the ternary eutectic or quasi-peritectic transformation.

## 3. Results

### 3.1. Calculation of Phase Diagram and Experimental Verification

#### 3.1.1. Calculation of Phase Diagram

**Figure 2:**
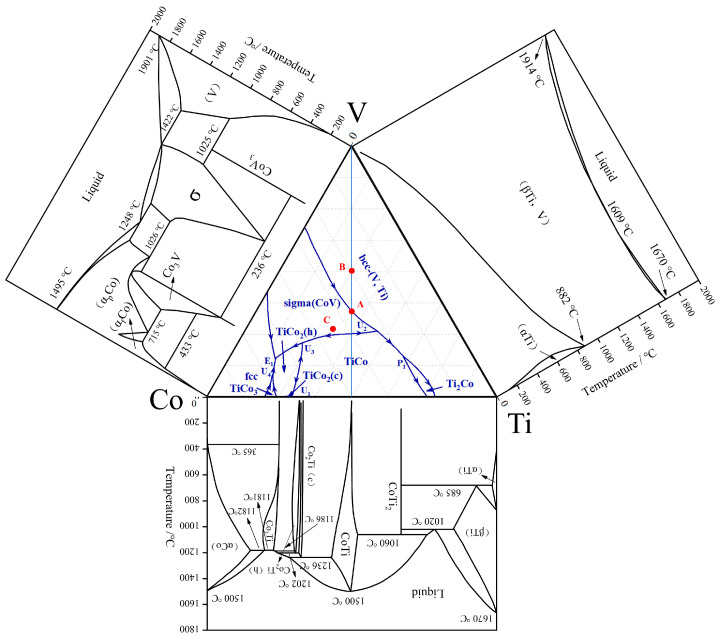
Calculated Co-V, V-Ti, and Ti-Co phase diagram and the calculated liquidus projection of the V-Ti-Co system. A, B and C represent the composition of V_35_Ti_32.5_Co_32.5_, V_50_Ti_25_Co_25_ and V_30_Ti_27_Co_43_, respectively.

Figure 2 shows the calculated Co-V, V-Ti, and Ti-Co phase diagrams as well as the calculated liquidus projection of the V-Ti-Co system over the whole composition using the presently obtained thermodynamic parameters in Table 1. Clearly, the Co-V binary system contains three intermediate phases, i.e., Co_3_V, CoV_3_, and σ-Co_2_V_3_. The last two phases are formed through the following reactions L + (V) → σ-Co_2_V_3_ (1695 K) and σ-Co_2_V_3_ + (V) → CoV_3_ (1297 K). The bcc solid solution (βTi, V) phase changed to the αTi phase at temperatures below 1073 K in the V-Ti binary system. Additionally, there are five intermediate phases (CoTi_2_, CoTi, Co_2_Ti (c), Co_2_Ti (h), and Co_3_Ti) in the Ti-Co binary system, which are formed through the following four reactions: (1) L → CoTi (1773 K); (2) L + CoTi → Co_2_Ti (c) (1509 K); (3) L + Co_2_Ti (c) → Co_2_Ti (h) (1475 K); and (4) L + Co_2_Ti (h) → Co_3_Ti (1459 K). All these thermodynamic data and equilibrium reactions are consistent with the results previously calculated by Bratberg [26], Okamoto [27], and Davydov [28].

The calculation of binary equilibrium is relatively simple. Nevertheless, the results of ternary alloys are more complex. Firstly, six invariant points, i.e., U_1_, U_2_, U_3_, U_4_, P_1_, and E_1_, exist in the V-Ti-Co liquidus curve, and details about these four-phase invariant reactions are summarised in Table 2. The arrows at the univariant lines designate declining temperatures. Secondly, due to the presence of univariant lines, the whole V-Ti-Co ternary phase diagram was divided into six single-phase regions, which are bcc-(V, Ti), TiCo, Sigma (CoV), fcc, TiCo_2_ (h), TiCo_2_ (c), TiCo_3_, and Ti_2_Co. Among them, the bcc-(V, Ti) and TiCo phase regions are the largest, and the univariant line connecting these regions provides convenient conditions for the subsequent development of dual-phase {bcc-(V, Ti) and TiCo} alloys. 

**Figure 3:**
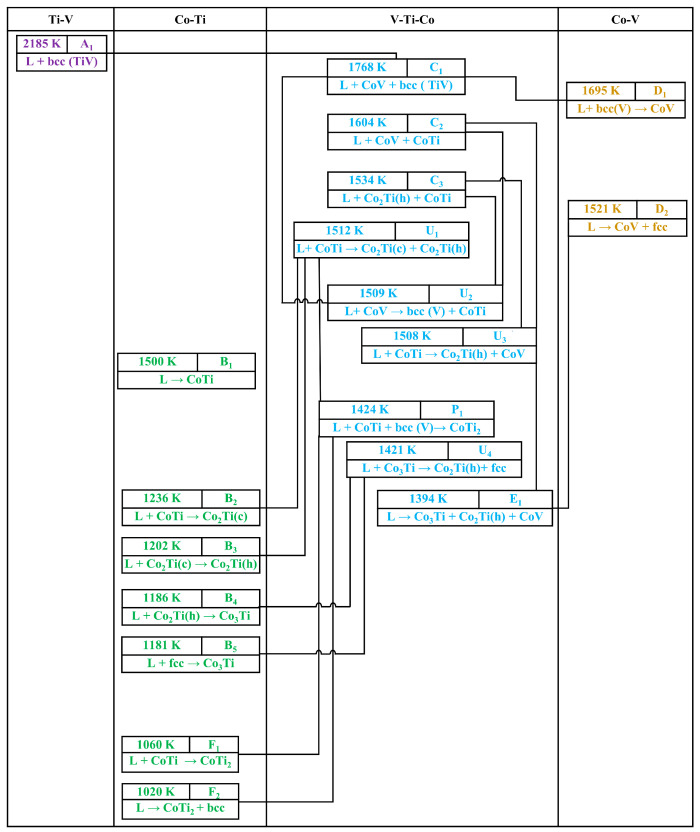
The reaction scheme of the V-Ti-Co system according to the present calculations.

Based on the results of the liquidus projection and invariant reactions mentioned above, the reaction scheme for the V-Ti-Co system was constructed for the first time, as shown in Figure 3.

The alloy solidifies and crystallises from the liquid phase at 1768 K until the end of the ternary eutectic reaction, L → Co_3_Ti + Co_2_Ti + CoV. As the temperature decreases, the other five isothermal equilibrium solidification reactions experienced in sequence are as follows:

U_1_: (L + TiCo → TiCo_2_ (h) + TiCo_2_ (c)), 1512 K;
U_2_: (L + sigma (CoV) → bcc − (V, Ti) + TiCo), 1509 K;
U_3_: (L + TiCo → TiCo_2_ (h) + sigma (CoV)), 1508 K;
U_4_: (L + TiCo_3_ → TiCo_2_ (h) + fcc), 1421 K;
P_1_: (L + TiCo + bcc − (V, Ti) → Ti_2_Co), 1394 K.

At these stages, the temperature remains constant, and three different solid phases will participate in the solidification and crystallization process, except for liquid alloys according to the phase diagram theory *F* = *P* + *C* + 2 (or 1 if *P* constant). In the isothermal sections of this system previously reported by Ruan et al. [15], Co_3_Ti was generated at 800 °C, while on the contrary, this phase was not found at 1000 °C. Additionally, there is very little remaining liquid phase on the isothermal sections at 1200 °C. These findings can be further explained through the phase diagram constructed in this work. The quasi-peritectic transformation of U_1_ and U_4_ consumes a large amount of the liquid phase and is accompanied by the formation of TiCo_3_, as shown in Figure 3. In brief, the above calculation results provide a better understanding of the solidification sequence and liquidus surface in the V-Ti-Co system.

#### 3.1.2. Experimental Verification

**Figure 4:**
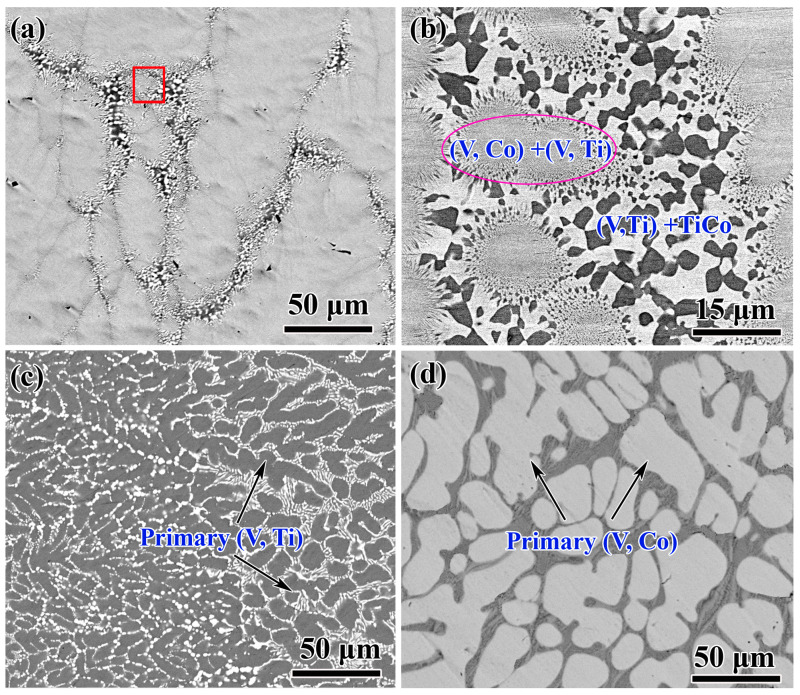
SEM images of the as-cast V-Ti-Co alloys: (**a**,**b**) V_35_Ti_32.5_Co_32.5_; (**c**) V_50_Ti_25_Co_25_; and (**d**) V_30_Ti_27_Co_43_. The right-side photograph in (**b**) is an enlargement of the red box areas in (**a**).

To verify the accuracy of the calculation results, three as-cast alloys with different compositions (see the marked positions A, B, and C in Figure 2) were prepared, and their microstructural characteristics are shown in Figure 4. According to the calculated phase diagram, the component of alloy A is located in the eutectic univariant line. Therefore, it can be inferred that eutectic reactions occur preferentially during its solidification process, generating eutectic structures. After observing and analysing the microstructure of alloy A, it is clear that it is mostly composed of homogeneous and fine eutectic {(V, Co) + (V, Ti)} structures (Figure 4a). At its edges, a small amount of coarse {(V, Ti) + TiCo} eutectic exists, see Figure 4b, which can be attributed to the quasi-peritectic equilibrium reaction of U_2_, L + sigma (CoV) → bcc-(V, Ti) + TiCo. For alloys B and C, due to their component being located in different phase regions, i.e., (V, Ti) and (V, Co), different primary phases will be generated during the solidification process. This assumption is confirmed by the results of Figure 4c,d, as the primary (V, Ti) and (V, Co) phases are clearly observed in their structure. The consistency between the calculated results and the experimental results further demonstrates the accuracy of the phase diagram calculation in the present work.

### 3.2. Solidification of V_x_Ti_50_Co_50−x_ Alloy

**Figure 5:**
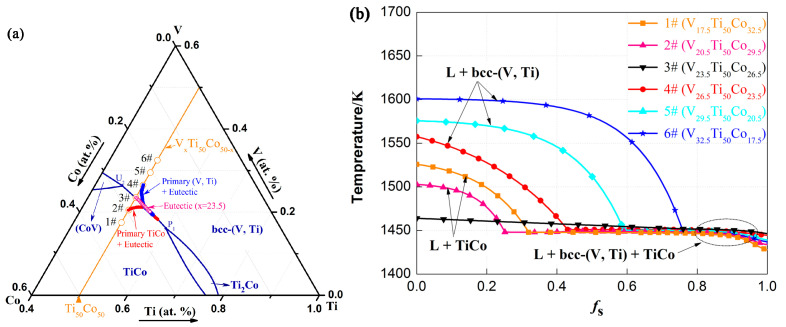
Calculated solidification paths of V_x_Ti_50_Co_50−x_ (x = 17.5, 20.5, …, 32.5) alloys: (**a**) the local liquid surface projection with the representative alloys 2#, 3#, and 4#; and (**b**) f_s_ vs. T for the selected alloys 1#…6#.

In our previously reported Nb-Ti-Co or Nb-Ti-Ni system [29,30,31], the duplex alloys composed of bcc-Nb solid solution and the B2-TiCo (or TiNi) compound were generally located on the Nb-TiCo and Nb-TiNi pseudo-binary isopleth, and these components are more suitable candidates for hydrogen permeable alloys. However, this pattern is not applicable in the V-Ti-Co system. For example, in addition to (V, Ti) and the TiCo phases, other phases, such as (V, Co) and Ti_2_Co additionally appear in as-cast V_35_Ti_32.5_Co_32.5_ (A) and V_50_Ti_25_Co_25_ (B) alloys, although these two alloys are both located in V-TiCo pseudo-binary system. Therefore, alloys in the quasi-binary isopleth are no longer considered. According to the calculated V-Ti-Co phase diagram in Figure 2, it is possible to obtain a bcc-(Nb, Ti) and TiCo dual-phase alloy at the lower right corner of the phase diagram, i.e., at a higher Ti content. So, a series of alloys with the formula V_23.5+x_Ti_50_Co_26.5−x_ (x = −6, −3, 0, 3, 6, 9) was devised and prepared, as shown in Figure 5a. The reason why Ti content remains at 50 at.% in the alloys is that the composition of this alloy series is close to the eutectic valley, avoiding the formation of impurity phases. Here, for the convenience of further discussion, these six alloys are named 1# to 6# in sequence, see Table 3 and Figure 5.

Before conducting the experiment, simulation calculations were first conducted on the solidification paths of alloys 1#–6#. Clearly, the solidification path for alloys 1# and 2# mainly includes the following two reactions: primary TiCo phase solidification (L→TiCo) and binary eutectic solidification [L→bcc-(V, Ti) + TiCo] (Figure 5b). Unlike the above alloys, primary bcc-(V, Ti) phases are formed by solidification before binary eutectic (bcc-(V, Ti) + TiCo) crystallization for alloys 4#, 5#, and 6#. Alloy 3# (V_23.5_Ti_50_Co_26.5_), as an exception, only undergoes the latter eutectic transformation with the formation of a eutectic microstructure. The above results indicate that ideal dual-phase structures can be obtained in these alloys. Nevertheless, the large temperature gradient in alloys 4#, 5#, and 6# suggests that the liquid phase surface of bcc-(V, Ti) is steeper and accompanied by residual stress and defect generation during its solidification process. In this case, cracks are easily formed inside the alloy, causing membrane separation failure.

### 3.3. Microstructure of V_x_Ti_50_Co_50−x_ Alloys

**Figure 6:**
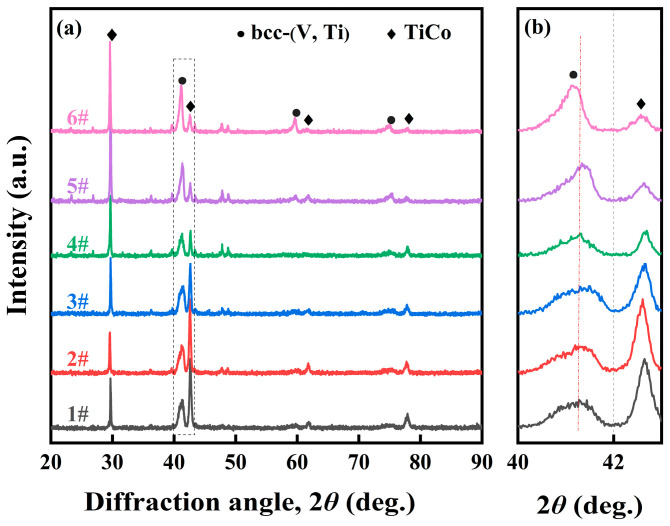
XRD patterns of V_x_Ti_50_Co_50−x_ alloy samples (1#…6#) (**a**), and locally enlarged peaks of the bcc-(V, Ti) phase (**b**).

Figure 6 shows the XRD spectrum of V_x_Ti_50_Co_50−x_ alloys (1#...6#). Through careful comparison, it can be found that these alloys are all composed of bcc-(V, Ti) and TiCo phases, except for a very small amount of undefined impurity phases. With an increase in the V element, the main diffraction peak intensity of the bcc-(V, Ti) phase increases, indicating that the volume fraction of this phase gradually increases. Furthermore, the position of this diffraction peak shifted to the left by varying degrees (see Figure 6b). Based on Vegard’s law and the Rietveld method, their crystal lattice parameters were calculated, and the relevant results are shown in Table 4. Obviously, the lattice parameters increase with the increase in V content, and 6# (V_23.5_Ti_50_Co_26.5_) has the largest lattice parameter of 3.791 Å. This may be related to the solid solution of Ti atoms in V. As the V content increases, more Ti atoms dissolve inside the lattice. Since the atomic radius of Ti (0.146 nm) is larger than that of V (0.132 nm), it causes V lattice expansion.

**Figure 7:**
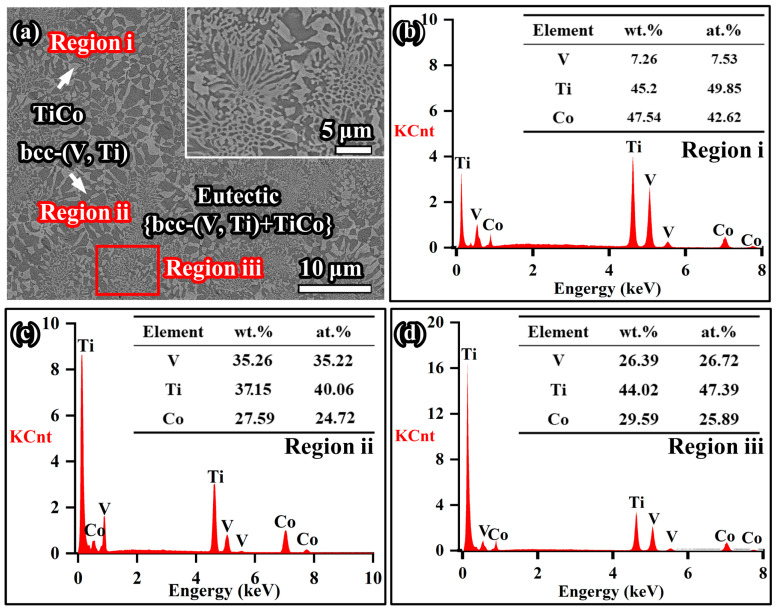
EDS results of the chemical compositions for each phase in eutectic V_23.5_Ti_50_Co_26.5_ (3#): (**a**) SEM micrograph; (**b**–**d**) represent the chemical compositions of TiCo, bcc-(V, Ti), and eutectic phases, respectively.

These alloys were further analysed by EDS and SEM, and the EDS result of the representative 3# alloy is shown in Figure 7. Clearly, it is mainly composed of two phases. The ratio of Ti/Co for the grey phase is approximately 1:1, and thus it is inferred as TiCo (Figure 7b). Using similar compositional analysis methods, the black phase is identified as bcc-(V, Ti), and up to 40 at.% of the Ti atoms are solidly dissolved in the V matrix (Figure 7c). The combination of the above two phases together forms a eutectic {bcc-(V, Ti) + TiCo} microstructure, with a composition of V_26.72_Ti_47.39_Co_25.89_, similar to that of alloy 3# (V_23.5_Ti_50_Co_26.5_) (see Figure 7d). In addition, as the V content increases, the content of solid solution Ti elements in the primary bcc-(V, Ti) phase of the alloy gradually increases (see Table 3). 

**Figure 8:**
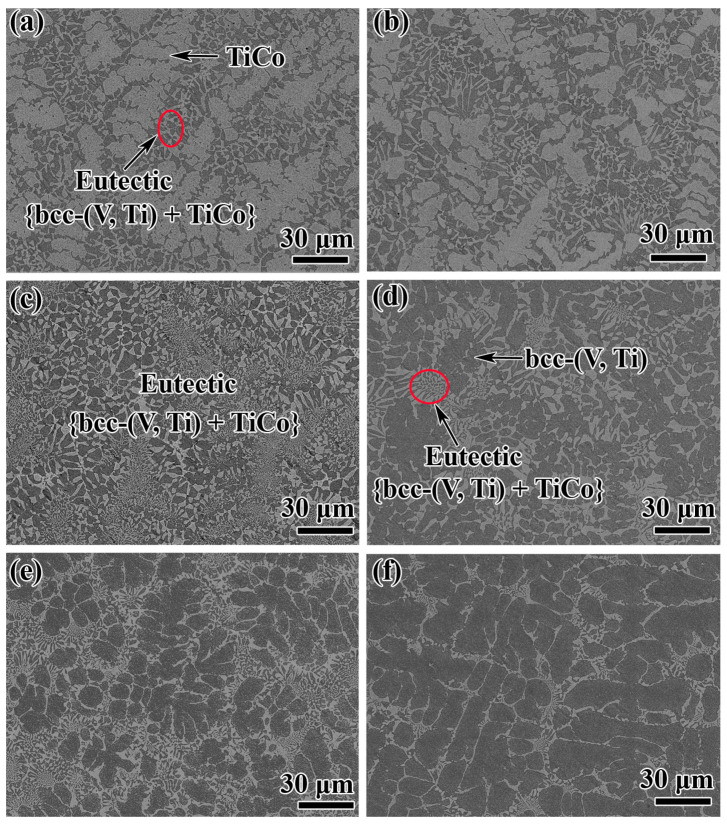
SEM images of the as-cast V_x_Ti_50_Co_50−x_ alloy samples (1#…6#): (**a**) V_17.5_Ti_50_Co_32.5_ (1#), (**b**) V_20.5_Ti_50_Co_29.5_ (2#), (**c**) V_23.5_Ti_50_Co_26.5_ (3#), (**d**) V_26.5_Ti_50_Co_23.5_ (4#), (**e**) V_29.5_Ti_50_Co_20.5_ (5#), and (**f**) V_32.5_Ti_50_Co_17.5_ (6#).

The microstructures of all the alloys are shown in Figure 8. As expected, alloys 1# and 2# are composed of a primary TiCo phase and a eutectic {bcc-(V, Ti) + TiCo} microstructure (Figure 8a,b). On the contrary, the primary phase in the alloys 5#–6# is bcc-(V, Ti) (Figure 8d–f). An almost fully eutectic structure is observed in 3#, and the rod-shaped morphology gradually coarsens from the centre to the surrounding area (Figure 8c). These results suggest that as the V content increases, the TiCo phase gradually decreases, followed by the formation of a fully eutectic structure in the alloy. As the V content further increases, the bcc-(V, Ti) phase solidifies and increases. The main reason for these structural changes is that the studied alloy composition gradually crosses the univariant lines, U_2_P_1_, as illustrated in Section 3.1.1. Furthermore, Table 3 summarises the constituting phases of these alloys. By comparing with the calculated results, all experimental data are basically consistent with the calculation results, further demonstrating the accuracy and reliability of the calculation in this work. Overall, we successfully obtained a V-Ti-Co alloy with a dual-phase structure through a combination of simulation and experimental methods. Next, the hydrogen transport performance of these alloys is further analysed and characterised.

### 3.4. Hydrogen Transport Performance of V_x_Ti_50_Co_50−x_ Alloys

**Figure 9:**
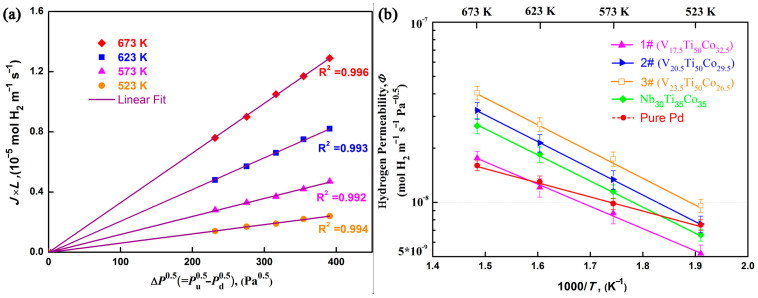
Hydrogen permeation performance of V_x_Ti_50_Co_50−x_ alloys (1#…6#): (**a**) representative relation between (J × L) and ΔP^0.5^ for the as-cast V_23.5_Ti_50_Co_26.5_ (3#) alloy; (**b**) temperature dependence of hydrogen permeability for samples 1#…6#, Nb_30_Ti_35_Co_35_ [11] and pure Pd [5,32].

Figure 9 shows the hydrogen permeability of the V_x_Ti_50_Co_50−x_ (1#…6#) alloys. To ensure the reproducibility of the results, each sample was measured at least twice during the hydrogen permeation experiment. When the error between two H_2_ flux values is less than 1%, their average value is calculated and substituted into Equation (1) to obtain the hydrogen permeability, wherein a good linear relationship between (*J* × *L*) and Δ*P*^0.5^ can be observed for the as-cast V_23.5_Ti_50_Co_26.5_ (3#) alloy (see Figure 9a). Similar relationships also exist for other alloys [11,32]. This suggests that the hydrogen permeation process through the membrane follows Equation (1) within the temperature range of 523–673 K, and this process is controlled by the bulk diffusion process rather than the membrane surface reaction. After linear fitting of each curve, the hydrogen permeability of each alloy at different temperatures was obtained, as shown in Figure 9b. Clearly, as the V content increases, the TiCo phase gradually decreases, but the hydrogen permeation performance increases. Interestingly, when the V content exceeds 23.5 at.%, the membrane experiences severe hydrogen embrittlement, and its hydrogen permeation performance cannot be measured. In this case, eutectic V_23.5_Ti_50_Co_26.5_ (3#) processes the highest hydrogen permeability among all studied alloys. Its maximum value is 4.05 × 10^−8^ mol H_2_ m^−1^ s^−1^ Pa^−0.5^ 673 K, which is 1.5 (2.5) times that of Nb_30_Ti_35_Co_35_ [11] (pure Pd [32]) and is also the highest value known heretofore in a V-Ti-Co system. 

From the slope of the Arrhenius equation (Figure 9b), the activation energy for hydrogen permeation through V-Ti-Co membranes was calculated, ranging from 23.39 to 23.57 kJ mol^−1^. Generally, the temperature and/or intermetallic diffusion has a significant impact on the formation of hydride phases in Nb- or V-based alloys. For example, as reported by Belyakova et al. [33], the critical temperature for β-hydride formation in Nb-Ti-Ni ternary alloys is 673 K. With the decrease in Ni and Ti contents in Nb and V, the critical formation temperature gradually decreases. Furthermore, it was found in Nb_15.6_Hf_42.2_Ni_42.2_ alloy [34] that the temperature for the formation of alloy hydride phase was lower, approximately 523 K. The increase in temperature promotes the formation of alloy hydrides. Similarly, the diffusion of solute atoms increases with the increase in temperature, which is also prone to the formation of hydride phases. Due to the integrity of the membrane (Figure 9) throughout the entire testing period, it can be inferred that the formation temperature of alloy hydride phase in V-Ti-Co alloys is higher than 673 K. These results also confirm, for the first time, that the V-Ti-Co alloys can be used as potential candidates for hydrogen separation and purification. 

**Figure 10:**
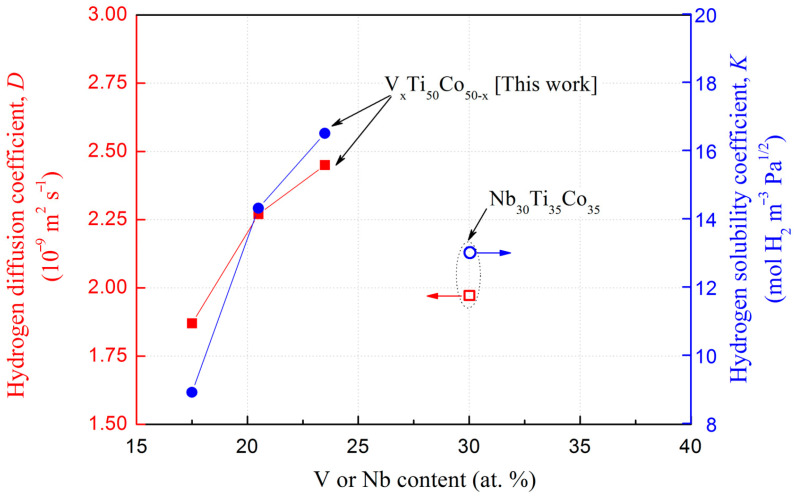
Dependence of hydrogen solubility and diffusivity on the V or Nb content for V_x_Ti_50_Co_50−x_ alloys (1#…6#) and Nb_30_Ti_35_Co_35_ [11,39] at 673 K.

By using the time lag method [35,36], the hydrogen diffusivity, *D*, values were calculated for hydrogen permeable alloys 1#–3#. Correspondingly, their hydrogen solubility, *K*, was also obtained through the formula *Φ* = *D* × *K*, as shown in Table 5 and Figure 10. On one hand, both *D* and *K* values increase with the increase in V content, indicating that the simultaneous increase in these two parameters leads to an increase in hydrogen permeability, as illustrated in Figure 9. One of the reasons for the gradual increase in hydrogen solubility is the increase in lattice parameters of the solid solution (V, Ti) phase, as shown in Table 4 above, because an increased octahedral or tetrahedral gap will provide more positions for hydrogen atoms [37]. On the other hand, compared to hydrogen diffusivity, hydrogen solubility has a higher rate of increase, and the above trend is more obvious when the V content is greater than 20 at.%. Furthermore, eutectic V_23.5_Ti_50_Co_26.5_ (3#) with lower V content has higher *K* and *D* values than eutectic Nb_30_Ti_35_Co_35_ with higher Nb content, indicating that the main element (i.e., 5B group element) is not the only element determining the permeability. Furthermore, the activation energy of hydrogen diffusion in 5B group alloys increases with the increase in hydrogen concentration [38]. Therefore, under the hydrogen permeation conditions in this work, a higher activation energy results in a decrease in the hydrogen diffusion coefficient of the studied alloys, which is lower than that of pure Pd. To a large extent, the microstructure, phase morphology, and solid solution elements all have important effects on the hydrogen permeation performance, which will be discussed in Section 4.

**Figure 11:**
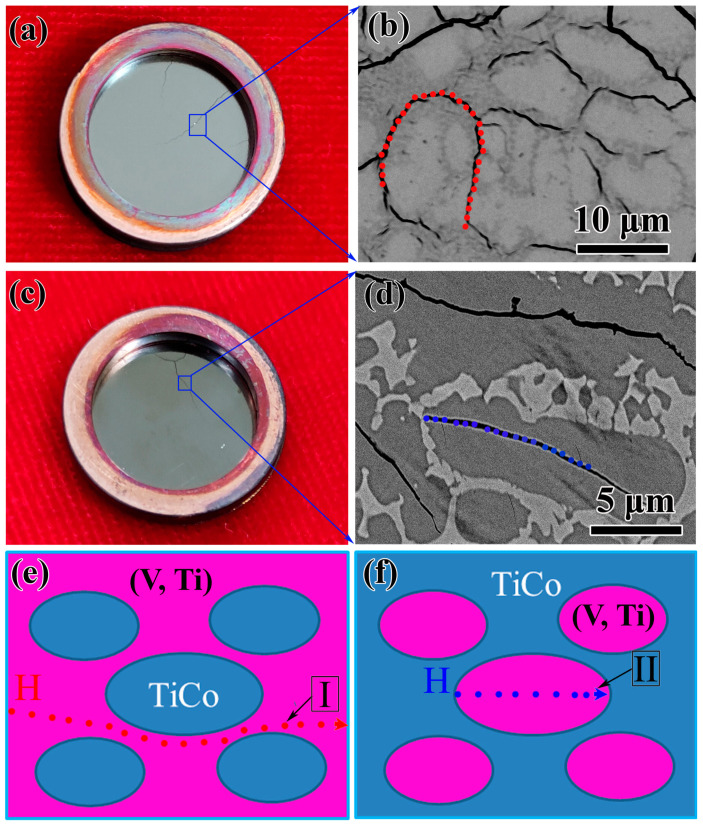
Hydrogen embrittlement fracture characteristics of V_x_Ti_50_Co_50−x_ alloys (1#…6#): (**a**,**c**) are the appearance of the feed-side of V_17.5_Ti_50_Co_32.5_ (1#) and V_29.5_Ti_50_Co_20.5_ (5#) membranes after permeation testing; (**b**,**d**) are the SEM images of cracked samples 1# and 5#; and (**e**,**f**) represent the schematic diagrams of hydrogen transport in alloys with different primary TiCo or bcc-(V, Ti) phases.

In addition, two different mechanical failure modes were found in V_x_Ti_50_Co_50−x_ (1#…6#) alloys, which are the intergranular crack failure mode and the intragranular crack failure mode (see Figure 11). The first mode is applicable for the alloys with a primary TiCo phase, whilst the latter is suitable for the alloys with a primary bcc-(V, Ti) phase. When the alloy is composed of a primary TiCo phase and a eutectic structure, such as alloy 1#, as shown in Figure 11a,b, the bcc phase in the eutectic provides a hydrogen atom diffusion channel, and cracks first appear in the eutectic structure, especially the bcc-(V, Ti) phase (Figure 11e). In this case, the bcc phase in the eutectic serves as the source of cracks and prioritises the initiation of cracks. On the contrary, for alloys containing a primary bcc-(V, Ti) phase, hydrogen atoms mainly penetrate or diffuse through this phase (path II), and the eutectic around this phase mainly plays a role in resisting hydrogen embrittlement. Thus, cracks are more likely to appear in the primary bcc-(V, Ti) phase (Figure 11c,d). In this situation, the presence of the eutectic effectively prevents intragranular cracking, as shown in Figure 11f. Overall, this study demonstrates that the fracture mode is closely related to the composition, an aspect not widely explored in 5B group alloys. Alloys with high V content (larger than 23.5 at.%) are affected by intergranular cracks, while intergranular cracks are more likely to form in samples with low Nb content (lower than 23.5 at.%) [40,41]. 

### 3.5. The Compositional Window Suitable for Hydrogen Permeation

**Figure 12:**
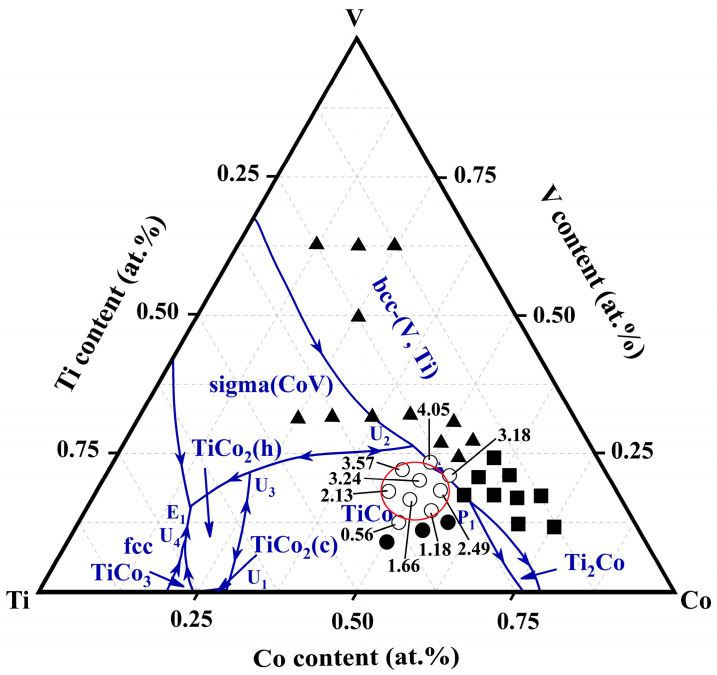
Hydrogen transport performance of the alloys investigated in this work plotted on the V-Ti-Co phase diagram. The solid triangles and squares represent brittle alloys, which exhibit hydrogen embrittlement fractures at room temperature and during the hydrogen permeation process. The circles indicate the alloys that can be used for the hydrogen permeation test, and the marked numbers are their Φ values at 673 K in the unit of 10^−8^ mol H_2_ m^−1^s^−1^Pa^−0.5^. The solid circles without marked values represent that their H_2_ flux is too low to measure by the mass flow meter.

To explore alloys with higher permeability, further research was conducted on 26 different alloys within the phase diagram, as shown in Figure 12. For ease of comparison, the parameters in these permeation experiments were consistent with those previously discussed in Section 3.4. After the series of tests and characterisations, these alloys can be divided into four categories based on their different hydrogen transport characteristics: low-temperature brittle alloys (▲), hydrogen brittle alloys (■), high-performance hydrogen permeable alloys (○), and alloys (●) with hydrogen permeability below 1 × 10^−8^ mol H_2_ m^−1^s^−1^Pa^−0.5^. The mechanical properties of the first two types of alloys are poor and cannot meet the requirements for membrane use. In the last category, their hydrogen permeability is too small to meet the requirements for hydrogen separation. Therefore, the range of components available for hydrogen permeation alloy design in the V-Ti-Co system is relatively narrow, only located near the lower left part of the univariant lines U_2_P_1_ (see the red ellipse in Figure 12). Of these alloys, the hydrogen permeability increases with an increase in the V content and Ti/Co ratio; eutectic V_23.5_Ti_50_Co_26.5_ (3#) possess the maximum value, 4.05 × 10^−8^ mol H_2_ m^−1^s^−1^Pa^−0.5^ at 673 K. Furthermore, this window is not located in or near the pseudo-binary V-TiCo isopleth, which is significantly different from Nb-Ti-Co or Nb Ti-Ni systems [42,43,44]. This finding breaks through the traditional limitations in the composition design of 5B group hydrogen permeable alloys, which requires the selection of composition at the position of the pseudo-binary isopleth.

## 4. Discussion

In a hydrogen-containing environment, almost all metal membranes face the problem of hydrogen embrittlement, including Pd-based membranes. This problem is exacerbated during the hydrogen atmosphere thermal cycle process. Doped alloy elements are an effective means of minimising the impact of embrittlement and helping to extend the membrane lifetimes. Common elements mainly involve Ti, Ni, Co, Hf, W, Mo, Cu, or a combination of them [39,45,46,47]. In this case, the key is to understand the phase diagram information of the multi-element alloy formed after doping elements and design reasonable components to avoid the generation of hydrogen-absorbing phases and hydride phase transition. So far, the challenge remains to construct a complete phase diagram of these alloy (quaternary or above) systems and obtain relevant thermodynamic information.

Alloys in the V-Ti-Co series are contradictory in terms of simultaneously increasing hydrogen embrittlement resistance and improving permeability. The reason for the above phenomenon is closely related to the composition of hydrogen permeable alloys, especially the Ti content in the (V, Ti) phase. As illustrated in Section 3.3, an increment in V content will cause more Ti atoms to solidly dissolve in the bcc-(V, Ti) phase. As reported by Ishikawa et al. [48], the diffusion coefficient of hydrogen in bcc-Ti (~1 × 10^−3^ cm^2^/s at 1173 K) is about one order of magnitude larger than that of bcc-V (~1.8 × 10^−4^ cm^2^/s at 1173 K). Therefore, increasing the solid solution of Ti atoms in the (V, Ti) phase is beneficial for increasing the hydrogen diffusion coefficient, thereby improving hydrogen permeability. For example, V_23.5_Ti_50_Co_26.5_ (3#), with higher solid solution Ti content, has higher permeability than V_17.5_Ti_50_Co_32.5_ (1#), as shown in Figure 9. 

Nevertheless, excessive Ti atoms will cause a higher hydrogen concentration in the membrane, posing a risk of hydrogen embrittlement. Ti is known to have a lower enthalpy of hydride formation compared to V, which makes the (V, Ti) phase, with more solid solution Ti atoms, more prone to form hydrides. Hydrides cause lattice expansion, resulting in membrane rupture. This phenomenon was also observed in V-Ti-Ni, Nb-TiCo and Pd-Ag alloys [49,50]. Therefore, controlling the Ti content in the alloy to be less than 50 at.% is crucial for preventing hydrogen embrittlement in a V-Ti-Co alloy system. Otherwise, hydrogen embrittlement will inevitably occur. In addition, the severe hydrogen embrittlement observed with V > 26.5 at.% alloys confirms that microstructural effects outweigh the effects of the Ti content in bcc-(V, Ti). The TiCo compounds, as well as eutectic structures, have a good role in resisting hydrogen embrittlement. Combined, these factors together co-determine the hydrogen transport properties of the bulk alloy. Furthermore, in our previous work about Nb-Ti-Co alloys [14], it was found that the impurities such as CO and CO_2_ significantly reduce the H_2_ flux at temperatures lower than 300 °C, while this situation improved at higher temperatures. Compared with inhibitor gases (CO, CO_2_), H_2_S showed greater effect on the permeability. This gas not only blocks H_2_ dissociation sites on the Pd surface, but also reacts with Pd to form brittle phases of Pd_4_S, causing membrane fracture. Similar situations may occur in V-TiCo membranes and further research is needed.

Although the high Ti content poses a risk of hydrogen embrittlement to the V-Ti-Co alloy membrane, the V_23.5_Ti_50_Co_26.5_ (3#) composed of a eutectic structure still exhibits high hydrogen permeation performance, far greater than the previously reported Nb-Ti-Co or Nb-Ti-Ni system. According to Ishikawa et al. [51], due to the position of the hydrogen permeable alloys in the pseudo-binary isopleth, the Ti content in the eutectic alloy of the Nb-Ti-Co (or Ni) system is relatively low, resulting in relatively low permeability. For example, the composition of the (Nb,Ti) phase in eutectic Nb_19_Ti_40_Ni_41_ is Nb_85_Ti_13_Ni_2_, and the ratio of Ti/Nb is merely 0.15. Correspondingly, its hydrogen permeability is only 0.8 × 10^−8^ mol H_2_ m^−1^s^−1^Pa^−0.5^ at 673 K. Similar cases can be found in eutectic Nb_30_Ti_35_Co_35_ alloys (Ti/Nb = 0.23). On the contrary, in the V-Ti-Co system, hydrogen-permeable alloys deviate from the position of the quasi-binary phase diagram near the Ti-rich angle region, as shown in Figure 12. This unique position endows the (V, Ti) phase in eutectic V_23.5_Ti_50_Co_26.5_ (3#) with a high Ti content, ~40 at.% (Ti/V = 1.14) (Figure 7). In comparison, dual-phase Nb-TiNi and Nb-TiCo alloys with the same high Ti content will inevitably precipitate primary bcc-Nb phases, further exacerbating the risk of hydrogen embrittlement in the membrane. In addition, due to the better ductility of eutectic alloy, the newly developed V-TiCo eutectic, i.e., V_23.5_Ti_50_Co_26.5_ (3#), in this work has great development value. If ultra-thin membranes are prepared by rolling or melt spinning, their H_2_ flux will be significantly increased. The relevant work is currently underway in our research group, which will be reported in the near future.

In addition, this article summarizes the advantages and disadvantages of V-Ti-Co alloys with other Pd-based ternary alloys used for hydrogen separation and purification. Compared with the Pd membrane, the newly developed V-Ti-Co alloys not only have the price advantage, but also exhibit higher hydrogen permeability. The price of a 25 μm-thick Pd is about USD 5000 per m^2^, while that of V-Ti-Co membrane costs merely ~ USD 200 per m^2^. Additionally, these alloy membranes can avoid hydride phase transition such as α→β in pure Pd, thus preventing the occurrence of hydrogen embrittlement. After surface coated with Pd, these dense membranes also exhibit good selectivity. However, it is undeniable that Pd-based alloy membranes still have unique advantages, i.e., excellent catalytic activity to dissociate hydrogen molecules into atoms. Although, an amorphous Zr_36_Ni_64_ membrane [52], not including noble Pd metals, can be used by itself for hydrogen permeation. However, the results about the permeation rate are not satisfactory. Furthermore, Pd-based membranes also have advantages in chemical tolerance in the presence of gaseous impurities (e.g., H_2_S) in the feedstock. For example, T.A. Peters and Way et al. investigated a variety of Pd-alloy membranes in the H_2_/H_2_S mixtures, and observed that Pd_85_Au_15_ foils show declines less in permeance compared with Pd_70_Cu_30_ or Pd alloys at 450 °C in 100 ppm H_2_S. Other Pd-based ternary alloys with excellent resistance to sulfur poisoning were reported by Haiyuan Jia [53], Fernando Braun [54], and Ana M. Tarditi [55]. If the above-mentioned Pd-based multi-element film is used instead of the original Pd film on the surface of Nb- or V-based alloy membrane, it is possible to improve the anti-sulfide poisoning phenomenon. However, further experiments are needed to confirm this.

In short, on one hand, this article constructs the equilibrium phase diagram of the V-Ti-Co system, providing guidance for subsequent experimental work using this phase diagram. On the other hand, some novel hydrogen permeation alloys, i.e., V_x_Ti_50_Co_50−x_ (x = 17.5–23.5), were explored, providing a reference for material selection in the field of membrane separation in the future.

## 5. Conclusions

In the present work, the equilibria phase diagram of the V-Ti-Co system was constructed by combining experimental and computational methods. Six ternary invariant reactions were confirmed to be located in the liquidus projection, and the phase diagram was divided into eight phase regions by their connecting lines. During solidification, three solid solution phases and five binary compound phases exist in this system. On the contrary, no ternary compound was found. In addition, some alloys, e.g., V_x_Ti_50_Co_50−x_ (x = 17.5–23.5), in the TiCo phase region, exhibit a high hydrogen permeability without hydrogen embrittlement. In particular, V_23.5_Ti_50_Co_26.5_ exhibits the highest permeability of 4.05 × 10^−8^ mol H_2_ m^−1^s^−1^Pa^−0.5^ at 673 K, which is the highest value known heretofore in a V-Ti-Co system. This higher permeability can be attributed to the simultaneous increment of hydrogen solubility and diffusivity, which is closely related to the high Ti and V content.

## Figures and Tables

**Table 1:** The thermodynamic parameters used in the present work.

Parameters		Values	Ref.
Solidification shrinkage		0.035	[20]
The distance of secondary dendrite (μm)		0.1	Calculated
V–Ti–Co	*D*_Co_ (mm^2^ s^−1^)	27 exp (−13,000/*T*)	Present work
	*D*_Ti_ (mm^2^ s^−1^)	22.3 exp (−11,000/*T*)	Present work
*L*_bcc-V_ (J mol^−1^)		−252,850 + 83*T* + (−8965 + 3.56*T*) × (*x*_V_ − *x*_Ti_)	Present work
*L*_Co2Ti_ (J mol^−1^)		−34,618 + 14.6*T* + (4583 − 62.8*T*) × (*x*_Co_ − *x*_Ti_)	Present work
*L*_TiCo_ (J mol^−1^)		35,782 + 1.67*T* + 2678 × (*x*_Ti_ − *x*_Co_)	Present work
*L*_CoTi2_ (J mol^−1^)		42,748 + 1.35T − 35.93 × (*x*_Ti_ − *x*_Co_)	Present work
*L*_Co3V_ (J mol^−1^)		−11,876 + 2.49*T* + 2781 × (*x*_Co_ − *x*_V_)	Present work
Solidification/cooling rates *R_f_* (s^−1^)		300	Calculated
Step length of α (Δ*f*_s_)		0.0025	Initial value
Step length of binary eutectic Δ*T* (°C)		0.25	Initial value
Specific heat (*S* and *L*) *c_PS_*, *c_PL_* (J kg^−1^K^−1^)		1068, 1241	[2,4,13]
Thermal conductivity (solid) *λ_S_* (W m^−1^K^−1^)		256	[2,4,13]
Thermal conductivity (liquid) *λ_L_* (W m^−1^K^−1^)		132	[2,4,13]
Liquidus temperature *T_liq_* (°C)		Depends on composition	By ThermoCalc

**Table 2:** Invariant reactions with liquid phases of V-Ti-Co system.

No.	Invariant Reaction	Reaction Type	Temperature (K)	Composition of Liquid Phases (at.%)		
				x (V)	x (Ti)	x (Co)
U_1_	L + CoTi → Co_2_Ti(c) + Co_2_Ti(h)	II	1512	29.423	0.201	70.376
U_2_	L + CoV → bcc (V) + CoTi	II	1509	45.571	26.462	27.967
U_3_	L + CoTi → Co_2_Ti(h) + CoV	II	1508	22.363	21.605	56.032
U_4_	L + Co_3_Ti → Co_2_Ti(h)+ fcc	II	1424	18.318	9.266	72.416
P_1_	L + CoTi + bcc (V)→ CoTi_2_	II	1421	59.761	16.297	23.942
E_1_	L → Co_3_Ti + Co_2_Ti(h) + CoV	I	1394	16.203	15.563	68.234

**Table 3:** The compositions, constituting phases, and the hydrogen permeability (*Φ*) of V_x_Ti_50_Co_50−x_ alloy samples (1#…6#).

No.	Samples	Constituting Phases	Chemical Composition of Primary bcc-(V,Ti)			Values of *Φ*, (mol H_2_ m^−1^s^−1^Pa^−0.5^)
			V	Ti	Co	
1#	V_17.5_Ti_50_Co_32.5_	TiCo, eutectic {bcc-(V,Ti) + TiCo}	—	—	—	1.66 × 10^−8^
2#	V_20.5_Ti_50_Co_29.5_	TiCo, eutectic {bcc-(V,Ti) + TiCo}	—	—	—	3.24 × 10^−8^
3#	V_23.5_Ti_50_Co_26.5_	Eutectic {bcc-(V,Ti) + TiCo}	—	—	—	4.05 × 10^−8^
4#	V_26.5_Ti_50_Co_23.5_	bcc-(V,Ti), eutectic {bcc-(V,Ti) + TiCo}	39.26	42.42	18.32	no permeation
5#	V_29.5_Ti_50_Co_20.5_	bcc-(V,Ti), eutectic {bcc-(V,Ti) + TiCo}	41.83	44.32	13.58	no permeation
6#	V_32.5_Ti_50_Co_17.5_	bcc-(V,Ti), eutectic {bcc-(V,Ti) + TiCo}	42.38	45.92	11.7	no permeation

**Table 4:** Crystal parameters of bcc-(V, Ti) phase in V-Ti-Co alloys.

No.	Samples	Lattice Parameters (Å)	Cell Volume (Å^3^)
1#	V_17.5_Ti_50_Co_32.5_	3.781	54.053
2#	V_20.5_Ti_50_Co_29.5_	3.784	54.187
3#	V_23.5_Ti_50_Co_26.5_	3.786	54.267
4#	V_26.5_Ti_50_Co_23.5_	3.788	54.354
5#	V_29.5_Ti_50_Co_20.5_	3.789	54.396
6#	V_32.5_Ti_50_Co_17.5_	3.791	54.483

**Table 5:** The values of hydrogen permeability, hydrogen solubility and hydrogen diffusivity for the V_x_Ti_50_Co_50−x_ alloy samples (1#…3#).

No.	Samples	Hydrogen Permeability	Hydrogen Solubility	Hydrogen Diffusivity
		[mol H_2_ m^−1^ s^−1^ Pa^−0.5^]	[mol H_2_ m^−3^ Pa^−0.5^]	[10^−9^m^2^ s^−1^]
1#	V_17.5_Ti_50_Co_32.5_	1.66 × 10^−8^	8.91	1.87
2#	V_20.5_Ti_50_Co_29.5_	3.24 × 10^−8^	14.3	2.27
3#	V_23.5_Ti_50_Co_26.5_	4.05 × 10^−8^	16.5	2.45
—	Nb_30_Ti_35_Co_35_ [11]	2.53 × 10^−8^	13.2	1.93
—	Nb_30_Ti_35_Ni_35_ [8]	1.55 × 10^−8^	32.55	0.48
—	Pd [5,32]	1.6 × 10^−8^	4.19	38.18
—	Pd_75_Ag_25_ [1]	3.21 × 10^−8^	—	—
—	Pd_60_Cu_40_ [2]	1.49× 10^−8^	—	—

## Data Availability

The raw/processed data required to reproduce these findings cannot be shared at this time as the data also forms part of an ongoing study.

## Nomenclature

*J*	hydrogen permeation flux [mol H_2_ m^−1^s^−1^]
*Φ*	hydrogen permeability [mol H_2_ m^−1^s^−1^Pa^−0.5^]
*L*	membrane thickness [m]
*P_u_*/*P_d_*	pressure difference of the upstream/downstream sides [Pa]
G*_i_*	Gibbs energy [kJ/mol]
$H_{i}^{SER}$	enthalpy of i under standard pressure [kJ/mol]
A–J	calculated constants.
R	gas constant
$x_{i}^{0}$	mole fractions of the pure element
$T_{ij}^{\varphi }$	the *i*th Redlich-Kister parameter
$T_{i}^{\varphi }$	the *i*th Curie temperature
